# PDF neuron firing phase-shifts key circadian activity neurons in *Drosophila*

**DOI:** 10.7554/eLife.02780

**Published:** 2014-06-17

**Authors:** Fang Guo, Isadora Cerullo, Xiao Chen, Michael Rosbash

**Affiliations:** 1 Department of Biology, Brandeis University, Waltham, United States; 2 National Center for Behavioral Genomics, Brandeis University, Waltham, United States; 3 Howard Hughes Medical Institute, Brandeis University, Waltham, United States; University of California, San Francisco, United States

**Keywords:** circadian rhythms, phase shift, PDF, locomotor activity, neuron activity, *D. melanogaster*

## Abstract

Our experiments address two long-standing models for the function of the *Drosophila* brain circadian network: a dual oscillator model, which emphasizes the primacy of PDF-containing neurons, and a cell-autonomous model for circadian phase adjustment. We identify five different circadian (E) neurons that are a major source of rhythmicity and locomotor activity. Brief firing of PDF cells at different times of day generates a phase response curve (PRC), which mimics a light-mediated PRC and requires PDF receptor expression in the five E neurons. Firing also resembles light by causing TIM degradation in downstream neurons. Unlike light however, firing-mediated phase-shifting is CRY-independent and exploits the E3 ligase component CUL-3 in the early night to degrade TIM. Our results suggest that PDF neurons integrate light information and then modulate the phase of E cell oscillations and behavioral rhythms. The results also explain how fly brain rhythms persist in constant darkness and without CRY.

**DOI:** http://dx.doi.org/10.7554/eLife.02780.001

## eLife digest

Most animals have daily rhythms of activity: some are awake during the day and asleep at night, whilst others are more active at night, or during the twilight hours around dawn and dusk. These cycles of activity are driven by an internal body clock, which is reset in response to external cues, like light and temperature, and which keeps the animal in sync with the day–night cycle.

The fruit fly *Drosophila* has daily—or circadian—rhythms of behavior, which are controlled by a network of genes that are switched ‘on’ or ‘off’ at different times in every 24-hr period. These circadian genes encode various proteins, including PERIOD and TIMELESS. The levels of these two proteins increase during the day and into the night, until they reach a point at which they cause their own genes to be switched off. PERIOD and TIMELESS are then destroyed each morning, and the cycle begins anew. Most of these same proteins perform similar functions in mammals.

In the fly brain, two groups of neurons express these key proteins and control the timings of activity or movement. One group, called M cells, regulates activity in the morning and also produces a small molecule called PDF. Another group, called E cells, controls evening activity, but is less well-defined.

Since M cells can maintain circadian rhythms even in total darkness, these cells were also considered key ‘pacemaker neurons’. However, Guo et al. now challenge this view by identifying five E cells that are the major source of circadian activity. Blocking the release of signaling molecules from these neurons severely disrupted movement in both the morning and the evening. The E cells are also critical for timekeeping under a normal day–night cycle.

Guo et al. found that the rhythm of the E cells is reset when the M cell neurons fire, which causes a release of PDF molecules. Further, PDF molecules reset the E cells by causing the degradation of the TIMELESS protein—which is similar to the effect of light, although light cause TIMELESS to be degraded via a different biochemical pathway.

Guo et al. suggest that under normal light–dark conditions the E cells are important for driving the flies' activity as well as for overall timekeeping. The M cells, instead, appear to function primarily to integrate information about light and reset the E cell clock. Challenges moving forward will include understanding other ways in which the firing of neurons can affect timekeeping, as well as looking if there any differences between the five E cells.

**DOI:** http://dx.doi.org/10.7554/eLife.02780.002

## Introduction

Animals use endogenous circadian pacemakers to control their physiology and behavior with roughly 24-hr periodicity (Bass and Takahashi, 2010; Thut et al., 2012). Intracellular timekeeping mechanisms include transcriptional feedback loops, which involve many key genes in *Drosophila*. They include *period* (*per*), *timeless* (*tim*), *clock* (*clk*), *cycle* (*cyc*), and *doubletime* (*dbt*). Coordination of their encoded protein activities (*period*, PER; *timeless*, TIM; *clock*, CLK; *cycle*, CYC: and *doubletime*, DBT) contributes to the intracellular cycling of the molecular pacemaker, which is similar between flies and mammals (Dubruille and Emery, 2008; Menet and Rosbash, 2011). PER and TIM concentrations increase during the day, and they eventually negatively regulate their own transcription. Biochemical oscillations in the head occur in part through a direct interaction of PER and TIM with the positive transcription factor CLK: CYC (Dubruille and Emery, 2008; Menet and Rosbash, 2011). They also require the photoreceptor cryptochrome (CRY) as well as a cycling light:dark (LD) environment, that is, RNA and protein oscillations damp rapidly in constant darkness (Stanewsky et al., 1998). The central brain is probably different as its molecular and behavioral rhythms persist in constant darkness and is CRY-independent (Stanewsky et al., 1998).

Nonetheless, CRY is expressed within many of the 75 pairs of central brain circadian neurons and is necessary for an important biological feature of circadian rhythms, namely, light-mediated phase adjustment or phase-shifting (Emery et al., 1998). There is good evidence in favor of a cell-autonomous view of *Drosophila* phase-shifting: light penetrates the thin insect cuticle (Fogle et al., 2011) and causes a CRY conformational change within circadian neurons (Ozturk et al., 2011). The altered CRY molecules recruit the E3 ligase JETLAG (JET) to TIM (Koh et al., 2006; Peschel et al., 2009). Premature TIM degradation then causes phase advances in the late night, whereas inappropriate TIM degradation during the TIM accumulating phase in the early night causes phase delays.

Two groups of central brain circadian neurons appear particularly important for behavioral rhythms. The 4 PDF-expressing small ventrolateral neurons (sLNvs) dictate morning activity as well as the rhythmicity in constant darkness (Renn et al., 1999; Blanchardon et al., 2001; Stoleru et al., 2004). This latter feature, free running locomotor activity rhythms, has caused the s-LNvs to be considered the major fly pacemaker neurons. A less well-defined set of cells directs evening activity. These neurons (E cells) also dictate circadian behavior in constant light and probably include the 6 LNds and the PDF-negative 5^th^ s-LNv (Picot et al., 2007).

We identify here five E neurons as a major source of circadian and behavioral rhythmicity as well as locomotor activity, of which the 2 CRY+ E neurons are most important. The phase of these cells is shifted by the M cell firing and requires PDF as well as the PDF receptor within these five E cells. Moreover, brief firing of M cells at different times of day generates a phase response curve (PRC), which resembles a proper light-mediated PRC. Brief M cell firing also resembles light by causing rapid TIM degradation within downstream circadian neurons, but firing-mediated phase-shifting is CRY-independent and exploits the E3 ligase components CUL-3 to degrade TIM, at least in the early night-delay zone. Our findings suggest that E cells are very important for timekeeping under light–dark conditions whereas an important function of PDF cells is for light-mediated phase adjustment, that is, to integrate light information and appropriately fire. This degrades TIM within E cells, which modulates the phase of E cell oscillations and behavioral rhythms.

## Results

### Identification of an E cell subset as major circadian and locomotor activity neurons

**Figure 1.**
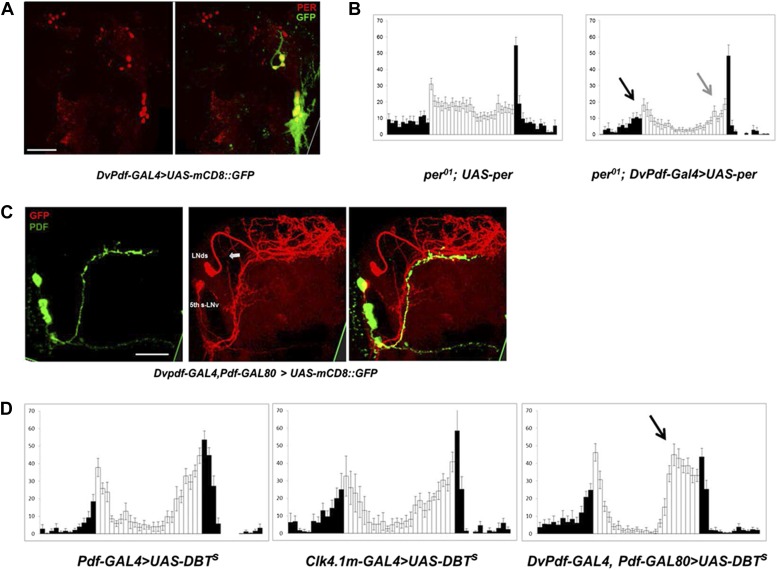
Characterization of the five E cells labeled by *DvPdf-GAL4, Pdf-GAL80*. (**A**) The brain expression pattern of *DvPdf-GAL4; UAS-mCD8::GFP* flies. Immunostaining with anti-PER only (red, left panel) and with anti-GFP (green) as well as anti-PER (right panel). GFP and PER co-localize in the PDF positive cells, LNds and the 5^th^-sLNv. The scale bar = 50 μm. A maximum intensity projection of confocal image stacks containing the cell bodies regions is shown. (**B**) Rescue of PER expression with *DvPdf-GAL4* in a *per*^*0*^ background restores both morning and evening anticipation peaks. *per*^*0*^ flies have no morning and evening anticipation peaks (left panel), whereas *per*^*0*^; *DvPdf-GAL4; UAS-per* flies show normal morning and evening peaks (right panel). Black and gray arrows point to morning and evening anticipation peaks, respectively. White and black bars indicate activity events/30 min bin during the day and night of the LD cycle. Error bars represent standard error of the mean (SEM). n = 15–16 for each group. (**C**) GFP immunostaining of *DvPdf-GAL4, Pdf-GAL80; UAS-mCD8::GFP* flies. GFP (red) is expressed in 3–4 LNds and the 5^th^-sLNv (middle panel), and PDF (green) is expressed in the large and small LNvs (left panel). The white arrow shows additional tracts from Dv-E cells that project to the accessory medulla (aMe). The scale bar = 50 μm. (**D**) Averaged group activity profiles from *UAS-DBT*^*s*^ expressed with *Pdf-GAL4, Clk4.1m-GAL4* and *DvPdf-GAL4, Pdf-GAL80* flies. The black arrow shows the advanced evening anticipation peak. Averaged over 2 days of LD data. White and black bars indicate activity events/30 min bin during the day and night of the LD cycle, respectively. Error bars represent standard error of the mean (SEM). n = 15–16 for each group. **DOI:** http://dx.doi.org/10.7554/eLife.02780.003

To address further the roles of M and E cells, we made use of a recently described *DvPdf-GAL4* driver, which expresses strongly and specifically in the M cells and subsets of the E cells. To verify this expression pattern, we crossed *DvPdf-GAL4* with *UAS-mCD8::GFP*. Exactly as reported (Bahn et al., 2009), GFP is expressed in the PDF-positive LNv cells and in several E cells in each hemisphere (Figure 1A); the latter consist of the single PDF-negative 5^th^-sLNv (Figure 1C) and 4 LNds (subsequently referred to as the 5 Dv-E cells). Cell identification of these circadian neurons was confirmed by co-staining with anti-PER antibodies (Figure 1A).

Rescuing PER expression with the *DvPdf-GAL4* driver restored both morning and evening anticipation to the arrhythmic *per*^*01*^ strain (Figure 1B), indicating an important role of these cells in circadian behavior. The five E cells labeled with *DvPdf-GAL4, Pdf-gal80* (henceforth called the *Dv-E* driver and the Dv-E cells) project to the dorsal brain and to the accessory medulla (aMe) as previously described (Figure 1C, arrows) (Bahn et al., 2009); the latter is based on a projection from the E cells, which contacts the M cells and is similar to that recently described with anti-ITP and anti-CRY antibodies(Yoshii et al., 2008; Johard et al., 2009).

To compare the contribution of different clock neurons to circadian timing under standard LD conditions, we overexpressed the period-altering kinase DBT^S^ (Muskus et al., 2007) under the control of three different circadian drivers. Although M cells (PDF neurons) are considered master pacemakers, neither expression of DBT^S^ in M cells nor in its direct downstream target neurons-DN1ps (Zhang et al., 2010a, 2010b; Seluzicki et al., 2014) changes the locomotor activity pattern or phase (Figure 1D). In contrast, the evening peak phase is dramatic advanced when accelerating the endogenous clock in the 5 Dv-E cells (Figure 1D), indicating that the E cells independently determine the major activity phase in LD (Stoleru et al., 2007).

**Figure 2.**
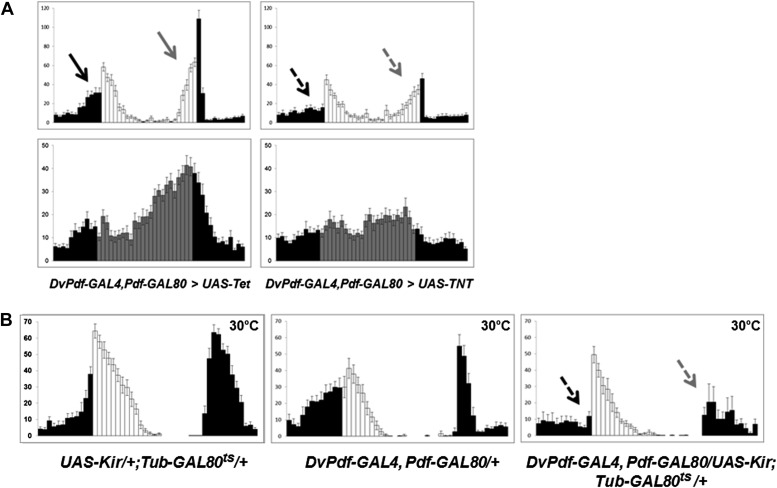
The five Dv-E cells are essential for circadian activity. (**A**) Group activity profiles during LD (top) and DD (bottom) cycles from *DvPdf-GAL4, Pdf-GAL80/UAS-Tet and DvPdf-GAL4, Pdf-GAL80/UAS-TNT* flies averaged over 3 days of LD or DD data. White/black bars, LD cycle; Grey/black bars, DD cycle. Error bars represent standard error of the mean (SEM) and n = 16 for each group. Arrows indicate morning anticipation (black) and evening anticipation (light gray), and dashed arrows indicate attenuated morning anticipation (black) and evening anticipation (light gray). The activity (with the standard error of the mean), DD rhythmicity and period are also shown. (**B**) Averaged group activity profiles during LD cycles from *UAS-Kir/+; TubGAL80*^*ts*^*/+* (left panel), *DvPdf-GAL4, Pdf-GAL80/+* (middle panel) and *DvPdf-GAL4, Pdf-GAL80/UAS-Kir; TubGAL80*^*ts*^*/+* (right panel) flies at 30°C. The left and middle panels are the two parental control strains and the right panel is the activity-inhibited strain. At the high temperature the control flies show a typical increased morning peak and delayed evening peak. In the right panel, the GAL80^ts^ become inactivated at 30°C allowing KIR expression in the E cells. The dashed arrows indicate attenuated morning and evening peaks. White/black bars indicate activity events in day/night as above. n = 24 for *DvPdf-GAL4, Pdf-GAL80/UAS-Kir; TubGAL80*^*ts*^*/+*flies and n = 20 for parental groups. Error bars represent SEM. **DOI:** http://dx.doi.org/10.7554/eLife.02780.004

Because differential neuron expression profiling suggests that the 5 Dv-E cells use excitatory neurotransmitter machinery to communicate with other neurons (unpublished data) (Johard et al., 2009), we blocked neurotransmitter release from 5 Dv-E cells by expressing the tetanus toxin light chain (TNT) (Sweeney et al., 1995). Compared to control flies, that is, flies that express inactive tetanus toxin UAS-Tet, *Dv-E GAL4/UAS-TNT* flies have a severe circadian locomotor activity deficiency. It includes a significant attenuation of morning anticipation as well as evening anticipation (Figure 2A, arrows), a reduced major activity peak in DD and high levels of arrhythmicity (Figure 2A; Table 1). The mean locomotor activity of *Dv-E GAL4/UAS-TNT* flies compared to control flies was 44% in LD and 45% in DD.

**Table 1.** Circadian behavior parameters of different genotypes under constant darkness (DD) **DOI:** http://dx.doi.org/10.7554/eLife.02780.005

Genotype	N	Rhythmic N	Percent rhythmic	Period (hr) ± S.D.	Power ±S.D.
*Pdf-GAL4/+*	15	14	93.3	24.3 ± 0.1	93 ± 15
*UAS-DBTs/+*	16	16	100	23.7 ± 0.3	125 ± 19
*Clk4.1m-GAL4/+*	16	14	87.5	24.2 ± 0.1	86 ± 15
*DvPdf-GAL4/+*	32	29	90.6	23.6 ± 0.2	143 ± 22
*DvPdf-GAL4,Pdf-GAL80/+*	30	26	86.7	24.5 ± 0.5	79 ± 14
*DvPdf-GAL4/+;Pdf-GAL80/+*	16	14	87.5	24.2 ± 0.3	113 ± 26
*Pdf-GAL4/+;UAS-DBTs/+*	16	11	68.7	19.7 ± 1.2	74 ± 16
*Clk4.1m-GAL4/UAS-DBT*^*s*^	15	12	80	23.1 ± 0.4	104 ± 23
*DvPdf-GAL4,Pdf-GAL80/+;UAS-DBT*^*s*^*/+*	16	14	87.5	23.5 ± 0.1	69 ± 7
*DvPdf-GAL4,Pdf-GAL80/UAS-TNT*	16	4	25	24.1 ± 0.5	35 ± 6
*DvPdf-GAL4,Pdf-GAL80/UAS-Tet*	16	14	87.5	24.3 ± 0.2	134 ± 32
*UAS-Kir/+;Tub-GAL80*^*ts*^*/+ (30°C)*	20	16	80	25.7 ± 1	105 ± 16
*DvPdf-GAL4,Pdf-GAL80/UAS-Kir;Tub-GAL80ts/+ (30°C)*	24	5	20.8	26.5 ± 2.2	27 ± 5
*DvPdf-GAL4,Pdf-GAL80/+ (30°C)*	20	15	75	24.1 ± 0.3	71 ± 13
*UAS-per*^*S*^*/+;Pdf-GSG/+ (RU-)*^**^**	15	15	100	23.3 ± 0.2	121 ± 12
*UAS-per*^*S*^*/+;Pdf-GSG/+ (RU+)*	16	10	62.5	20.5 ± 1.4	73 ± 15
*UAS-per*^*S*^*/UAS-Kir;Pdf-GSG/+ (RU−)*	14	13	92.9	23 ± 0.3	94 ± 13
*UAS-per*^*S/*^*UAS-Kir;Pdf-GSG/+ (RU+)*	16	3	18.7	22.8 ± 1.2	24 ± 6
*UAS-per*^*S*^*/+;Pdf-GSG/UAS-PDF RNAi (RU−)*	15	14	93.3	23.3 ± 0.2	88 ± 15
*UAS-per*^*S*^*/+;Pdf-GSG/UAS-PDF RNAi (RU+)*	16	2	12.5	22.3 ± 0.8	19 ± 3
*UAS-per*^*S*^*/+;Pdf-GSG/+ (RU+ to RU−)*	60	54	90	23.2 ± 0.1	94 ± 13
*UAS-per*^*S*^*/UAS-Kir;Pdf-GSG/+ (RU+ to RU−)*	64	41	64	23.6 ± 0.6	56 ± 11
*UAS-per*^*S*^*/+;Pdf-GSG/UAS-PDF RNAi (RU+ to RU−)*	62	35	56.5	23.8 ± 1.2	48 ± 7
*UAS-dTrpA1/+ (21°C)*	16	16	100	24.4 ± 0.4	117 ± 8
*DvPdf-GAL4/UAS-dTrpA1 (21°C)*	32	31	96.8	24.2 ± 0.3	93 ± 6
*DvPdf-GAL4,Pdf-GAL80/UAS-dTrpA1 (21°C)*	16	12	75	23.6 ± 0.5	72 ± 8
*Pdf-GAL4/UAS-dTrpA1 (21°C)*	32	31	96.9	24.2 ± 0.3	127 ± 15
*UAS-dTrpA1/+;TH-GAL4/+ (21°C)*	16	16	100	23.6 ± 0.4	166 ± 19
*per*^*S*^*;UAS-dTrpA1/+ (21°C)*	16	16	100	20.3 ± 0.2	131 ± 12
*per*^*S*^*;Pdf-GAL4/UAS-dTrpA1 (21°C)*	32	31	96.9	19.7 ± 0.6	102 ± 10
*per*^*S*^*;DvPdf-GAL4/UAS-dTrpA1 (21°C)*	32	31	96.9	20.1 ± 0.2	97 ± 13
*per*^*S*^*;DvPdf-GAL4,Pdf-GAL80/UAS-dTrpA1 (21°C)*	16	14	87.5	21.1 ± 0.7	125 ± 19
*pdfr*^*−*^*;UAS-PDFR*	16	6	37.5	21.9 ± 1.2	34 ± 7
*pdfr*^*−*^*;DvPdf-GAL4,Pdf-GAL80/UAS-PDFR*	18	13	72.2	24.8 ± 0.4	77 ± 10
*pdfr*^*−*^*;DvPdf-GAL4/UAS-PDFR;Cry-GAL80/+*	16	7	43.8	22.1 ± 0.7	37 ± 8
*pdfr*^*−*^*;DvPdf-GAL4,UAS-dTrpA1/UAS-PDFR (21°C)*	30	24	80	23.7 ± 0.9	69 ± 9
*pdfr*^*−*^*;DvPdf-GAL4,UAS-dTrpA1/UAS-PDFR;Pdf-GAL80/+ (21°C)*	24	18	75	23.8 ± 0.7	65 ± 13
*pdfr*^*−*^*;DvPdf-GAL4,UAS-dTrpA1/UAS-PDFR;Cry-GAL80/+ (21°C)*	24	11	45.8	21.5 ± 1	33 ± 5
*pdfr*^*−*^*;DvPdf-GAL4,UAS-dTrpA1/+ (21°C)*	32	13	40.6	21.2 ± 1.1	29 ± 8
*DvPdf-GAL4,UAS-dTrpA1/+;cry*^*01*^ *(21°C)*	32	25	78.1	23.2 ± 0.7	64 ± 14
*DvPdf-GAL4,UAS-dTrpA1/+ (21°C)*	30	24	80	24.1 ± 0.3	71 ± 13
*DvPdf-GAL4,UAS-dTrpA1/+;UAS-Cul-3 RNAi #1/+ (21°C)*	31	25	80.6	24.6 ± 0.3	85 ± 8
*DvPdf-GAL4,UAS-dTrpA1/UAS-Cul-3 RNAi #2 (21°C)*	32	27	84.4	24.2 ± 0.4	73 ± 9
*DvPdf-GAL4,UAS-dTrpA1/+;UAS-Cul-3 RNAi #3/+ (21°C)*	32	25	78.1	24.1 ± 0.3	82 ± 19
*UAS-Cul-3 RNAi #1/+ (21°C)*	16	16	100	24.1 ± 0.2	131 ± 11
*UAS-Cul-3 RNAi #2/+ (21°C)*	16	15	93.8	23.5 ± 0.2	103 ± 21
*UAS-Cul-3 RNAi #3/+ (21°C)*	16	15	93.8	23.7 ± 0.1	95 ± 13

Use of a different reagent, expression of the potassium channel Kir, to suppress neuronal activity in the 5 Dv-E cells also causes a significant reduction in both morning and evening anticipation as well as high percentage of DD arrhythmicity (Figure 2B, right, arrows and Table 1) compared to the two parental strains (Figure 2B, left and middle). The use of *Tub-GAL80*^*ts*^ and high temperature was to prevent Kir expression during development. The unusual level of nocturnal activity at high temperature (previously reported, Majercak et al., 1999) does not obscure identification of the morning and evening anticipation peaks (Figure 2B).

### PDF and neuronal activity are required for M cells to reset period in DD

**Figure 3.**
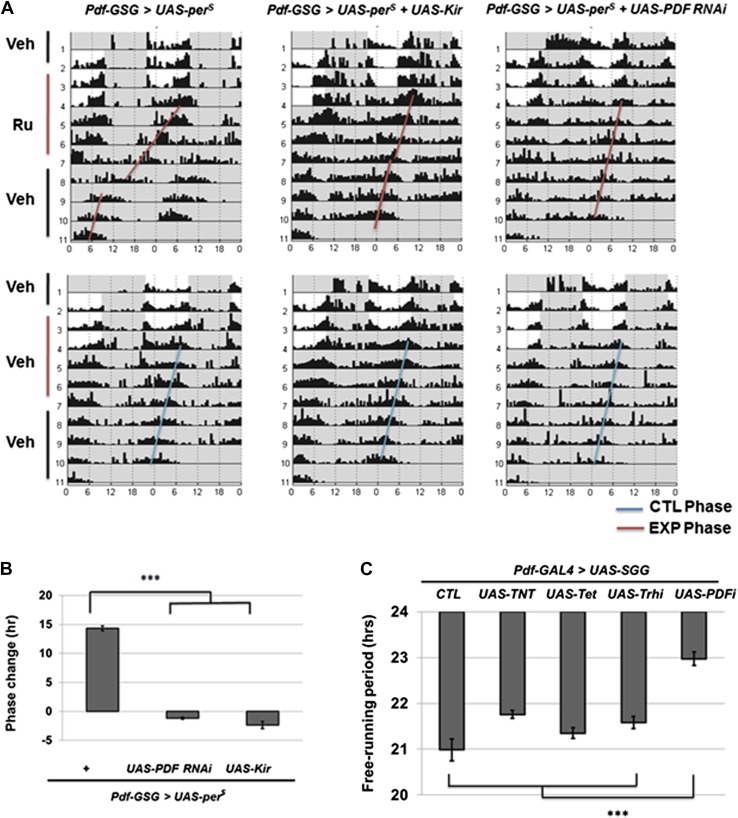
M cells use the PDF peptide and neuronal activity to adjust period in DD. (**A**) Accelerated M cells send PDF as a resetting signal to cause a daily advanced activity phase. Double plotted averaged actograms of representative individual flies from each genotype are shown. Experimental group were transferred to RU486 food for 2 days in LD and the maintained for 3 days in DD before being returned back to food containing vehicle. Control group were maintained in vehicle food. Flies expressing Kir and PDF RNAi in M cells gradually lose rhythmicity during the 3 drug feeding DD days. In the actograms, white background represents day, gray background represents darkness. Red lines indicate the DD phase of the experimental group, and the blue lines indicate the DD phase of control group. The genotype of each group was labeled above the panels. (**B**)The quantification of phase change for the genotypes described in (**A**). n = 60-64 for each group. ‘***’ means p<0.001 as determined by one way analysis of variance (ANOVA), Tukey post hoc test, and the error bars indicate SEM. (**C**) Co-expression of PDF RNAi in accelerates M cells prevented period shortening. The histogram shows the period of five genotypes: *UAS-SGG; Pdf-GAL4/+*, *UAS-SGG; Pdf-GAL4/UAS-TNT, UAS-SGG; Pdf-GAL4/UAS-Tet, UAS-SGG; Pdf-GAL4/UAS-Trh RNAi, UAS-SGG; Pdf-GAL4/UAS-PDF RNAi*. Tetanus toxin light chain (TNT) was used to block neurotransmitter releasing, and Tet is an inactive form of TNT, Trh RNAi was used as control of PDF RNAi since PDF cells do not express tryptophan hydroxylase (Trh), PDF RNAi is as in Figure 2B n = 13–15 for each group. ‘***’ represents p<0.001 as determined by one way analysis of variance (ANOVA), Tukey post hoc test, and the error bar indicates SEM. **DOI:** http://dx.doi.org/10.7554/eLife.02780.006

The M cells (s-LNvs) impact wild-type E cell molecular oscillations (Stoleru et al., 2005) as well as control DD rhythmicity. To identify relevant M cell signals, we repeated the previous strategy (Stoleru et al., 2005) but altered circadian period with M cell overexpression of *UAS-per*^*S*^; *per*^*S*^ is a missense mutation that causes a short period phenotype (Li and Rosbash, 2013). This was combined with simultaneous M cell expression of RNAi constructs to screen for the candidate signal molecule. Since long-term expression of RNAi constructs can have developmental effects, we used the *Pdf-geneswitch* system and fed flies with drug to activate GeneSwitch protein for 5 days , that is, 2 LD days and 3 DD days (Depetris-Chauvin et al., 2011). This protocol should temporally express PER^S^, which indeed shortened the circadian period to 20.5 hr (Figure 3A; Table 1). Since sibling flies on normal food maintained a normal 23.5 hr period and returning the flies to normal food also reversed the short period to 23.5 hr, short-term drug feeding is sufficient to induce the transgene and accelerate circadian period to generate an approximate 14 hr phase advance at the end of the protocol (Figure 3A,B).

Flies co-expressing PDF RNAi along with *UAS-per*^*S*^ showed no short period phenotype (Figure 3A,B). A series of control RNAi and tetanus toxin light chain (TNT) constructs suggests that the effect is specific to this single neuropeptide. For example, PDF cells may not express a classical neurotransmitter that causes E cells to follow accelerated PDF neurons (Figure 3C). Temporal co-expression of the inwardly rectifying potassium channel (Kir) with *UAS-per*^*S*^ similarly inhibited the short period phenotype, indicating that PDF cell firing is also required for maintaining PDF-mediated communication from M cells (Figure 3A,B). A recent paper also concluded that circadian period is determined by multiple independent oscillators, M cells and subset of E cells, which are coordinated by PDF signaling (Yao and Shafer, 2014). The data taken together indicate that PDF-cell firing and PDF generate the M cell resetting signal.

### Firing PDF positive M cells induces quasi-normal phase-shifts

The importance of M cell neuronal activity suggested that firing might be more generally relevant to phase resetting, despite the prevailing cell-autonomous model. To address this possibility, we artificially activated circadian neurons with the thermo-activated dTrpA1 cation channel under control of the *DvPdf-GAL4* driver and assayed the phase response. We used the well-established anchored PRC protocol (APRC; Kaushik et al., 2007) with firing induced by a 2 hr temperature shift of flies from 21°C to 30°C during the night of the last LD cycle; conditions were returned to 21°C and constant darkness for the rest of the experiment. We focused initially on a time in the early evening (ZT15) when M cell activity should be low (Cao and Nitabach, 2008; Cao et al., 2013) and more importantly when exposure to light (or even a 37°C heat pulse [Kaushik et al., 2007]) elicits a maximal phase-shift in wild-type flies.

**Figure 4.**
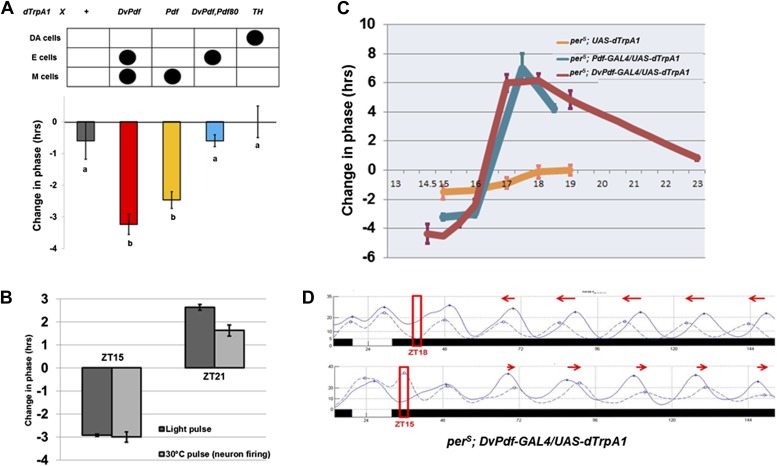
Activation of M cells is necessary and sufficient to trigger a phase-shift without light. (**A**) Only the drivers that express dTrpA1 in the PDF+ morning cells can cause a phase-shift in constant darkness. All of the *GAL4>UAS-dTrpA1* flies were first entrained during 3 LD days to synchronize their endogenous clock and the transferred to 30°C at ZT15 for 2 hr during the last LD night and then returned to 21°C for the following days in DD. GAL4 lines that exhibit substantial phase delays label the PDF-positive pacemaker neurons. n = 16–32 for each group. Genotype of each group was labeled above the histogram. DA = dopaminergic neurons. M and E cells were described in main text. The letters ‘a’ and ‘b’ indicate significantly different groups (p<0.001), by one way analysis of variance (ANOVA), Tukey post hoc test. The error bars indicate SEM. (**B**) Firing of PDF positive morning cells induces quasi-normal phase-shifts compared to light-induced phase-shifts. *Pdf-GAL4/UAS-dTrpA1 flies* exhibit a phase delay at ZT15 and a phase advance at ZT21 after a 2 hr 30°C pulse or a 2 hr light pulse. n = 32 for each group. The error bars indicate SEM. Note that phase-shift values are not very different from a standard 10 min light pulse. (**C**) The magnitude of a neuronal firing-induced phase-shift is larger in *per*^*S*^ flies. *per*^*S*^*; pdf-GAL4/UAS-dTrpA1, per*^*S*^*; DvPdf-GAL4/UAS-dTrpA1* and *per*^*S*^*; UAS-dTrpA1* flies were exposed to a 2 hr 30°C pulse at different circadian times. n = 16-32 for each group. The error bars indicate SEM. (**D**) The detailed phase shift panel of the 30°C pulse at ZT15 and ZT18 of *per*^*S*^*; DvPdf-GAL4/UAS-dTrpA1* flies. Red box indicates the time of the 2 hr pulse of 30°C. The dashed line represents the 30°C pulsed group and the solid line the control group. Red arrows indicate the direction of phase change. **DOI:** http://dx.doi.org/10.7554/eLife.02780.007

This protocol caused a phase delay of approximately 3–4 hr (Figure 4A), similar to the delay caused by a 2 hr saturating (2000 lux) light pulse at ZT15 (Bao et al., 2001). Addition of *Pdf-GAL80* to *DvPdf-GAL4*, that is, use of the *Dv-E* driver, had a dramatically reduced phase-shift, which indicates that PDF morning cell firing is necessary for the *DvPdf-GAL4* firing-induced phase-shift. Firing was next restricted to PDF neurons, that is, a 2-hr temperature shift of *Pdf-GAL4/UAS-dTrpA1* flies at ZT15. This protocol elicited a similar 3-hr phase delay (Figure 4A), indicating that PDF morning cell firing is not only necessary but also sufficient for a quasi-normal firing-mediated phase-shift at ZT15.

Firing of morning pacemakers also causes proper phase advances at ZT21, and control flies with TrpA1 expression in E cells but not in M cells show no phase advance (Figure 4B and data not shown). This indicates that PDF cell firing more broadly mimics light-induced phase-shifting. It is also notable that firing of many other neuronal subsets, including the strongly activity-promoting dopaminergic neuronal group with *TH-GAL4*, had little or no phase-shifting effect (Figure 4A and data not shown). These negative results confirm that a 2 hr 30°C pulse alone is unable to cause a substantial phase-shift.

To extend further this relationship, we assayed firing-induced phase-shifting in a *per*^*S*^ genetic background. This is because previous studies had shown that the *per*^*S*^ mutation not only shortens circadian period to 19 hr but also dramatically alters light-induced phase-shifting, from a low amplitude type 1 PRC to a high amplitude type 0 PRC (Bao et al., 2001). Similar to light, neuronal firing induced an exaggerated phase change in a *per*^*S*^ background compared to wild type in the advance zone (maximum 6–7 hr; Figure 4C). The phase-shifts of parental control strains without dTrpA1 expression were statistically indistinguishable from unheated groups, further indicating that the 2 hr 30°C pulse has no phase-shifting effect (Kaushik et al., 2007). Addition of *Pdf-GAL80* to the *per*^*S*^*; DvPdf-GAL4; UAS-dTrpA1* background dramatically reduced both advance and delay phase-shifts (data not shown), confirming that morning cell activity makes the major contribution to the generation of this enhanced phase-shift. We note that the ability of PDF to contribute to phase-shifting has been previously demonstrated in other insects (Petri and Stengl, 1997).

### PDFR function in Dv-E cells is sufficient to restore PDF mediated signaling

**Figure 5.**
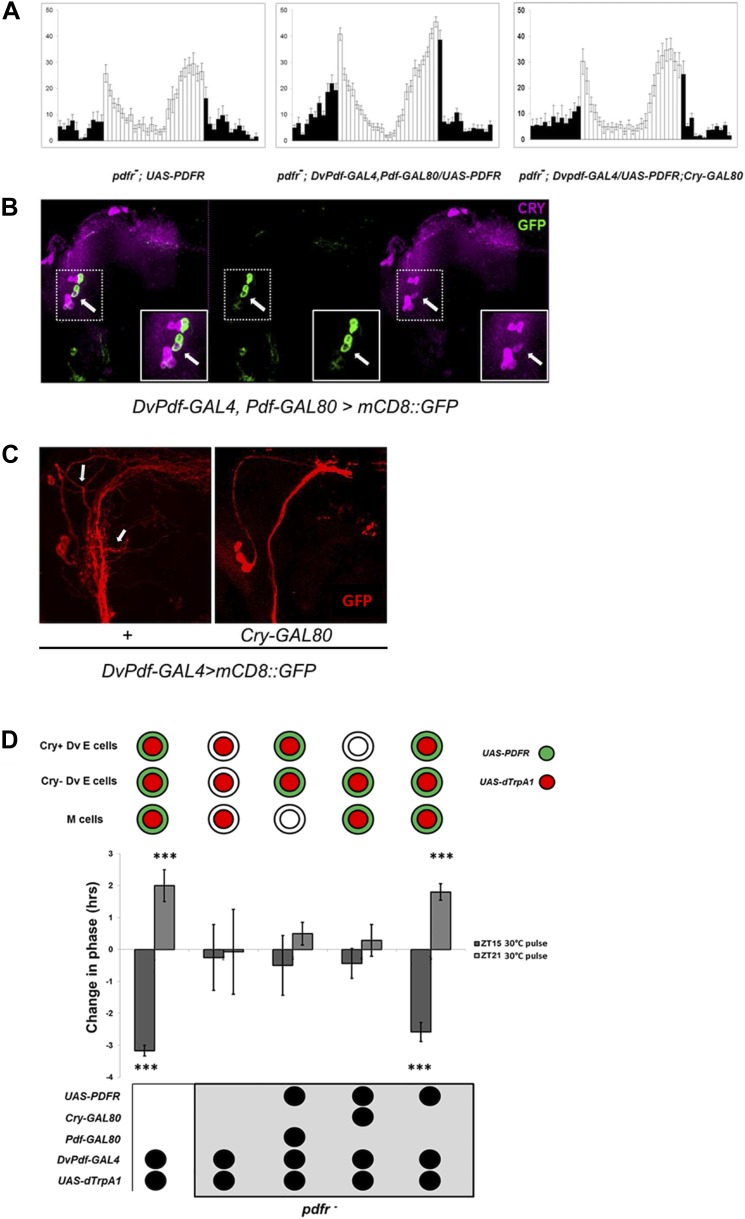
Restoring E cell PDFR rescues period, rhythmicity and firing-induced phase shifts. (**A**) *pdfr* mutant flies show no morning peak and an advanced evening peak as expected (left panel). Rescue of PDFR expression with the Dv-E cell driver in this mutant background restores both morning and evening anticipation peaks, that is, these flies show an intact morning peak and more normal onset of evening peak activity (middle panel). Inhibition of rescue in CRY-positive E cells with *Cry-GAL80* prevents the restoration of morning and evening activity peaks. n = 16–18 for each group. Genotypes are shown under each panel. The error bar indicates SEM. (**B**) CRY staining pattern in *DvPdf-GAL4, Pdf-GAL80; UAS-mCD8::GFP* brains. GFP (green) is expressed in 4 LNds (left and middle panel), and CRY signal (magenta) is visible in the large PDF cells (below and to the left of the arrow) as well as 3 LNds (left and right panel). The arrow shows the one CRY-positive DV-E cell. Magnified images of the upper dash line boxed area are shown in lower solid line box. (**C**) Adding a copy of *Cry-GAL80* eliminates GFP staining of the CRY-positive Dv-E cells as well as their branches, which are adjacent to the PDF cell dorsal projections. Brains from *DvPdf-GAL4/+; UAS-mCD8::GFP/+* (left panel) *and DvPdf-GAL4/+; Cry-GAL80/UAS-mCD8::GFP* (right panel) were stained with a GFP antibody (red). The upper arrow shows the typical projection from *DvPdf* -labeled CRY positive LNd neuron and the lower arrow points to likely branches from the 5^th^-sLNv. With *Cry-GAL80*, both of these projections are absent. Note that a copy of *Cry-GAL80* can block GAL4 activity in CRY positive Dv-E cells but not in sLNv, the dorsal projections from sLNvs are still visible in *DvPdf-GAL4/+; Cry-GAL80/UAS-mCD8::GFP* brains (right panel). (**D**) Flies were exposed to a 30°C pulse for 2 hr at ZT15 (dark gray bars) or ZT21 (light gray bars). The expression pattern of *UAS-dTrpA1* and *UAS-PDFR* is shown above the histogram. Genotypes shown below each histogram are (from left to right): *DvPdf-GAL4/UAS-dTrpA1* (n = 32), *pdfr*^*−*^*; DvPdf-GAL4/UAS-dTrpA1*(n = 32), *pdfr*^*−*^*; DvPdf-GAL4,UAS-dTrpA1/UAS-PDFR; Pdf-GAL80* (n = 24), *pdfr*^*−*^*; DvPdf-GAL4, UAS-dTrpA1/UAS-PDFR; Cry-GAL80* (n = 24) and *pdfr*^*−*^*; DvPdf-GAL4,UAS-dTrpA1/UAS-PDFR* (n = 30). ‘***’ represents p<0.001 as determined by the student's *t* test and indicates a significant phase change. The error bars are SEM. **DOI:** http://dx.doi.org/10.7554/eLife.02780.008

Given the important role of the Dv-E cells to circadian timing and to locomotor activity in DD as well as LD (Figures 1 and 2), we suspected that they are important downstream targets of PDF and therefore important sites of PDFR expression. Like *pdf*^*01*^ mutant flies, a mutant in PDFR (*pdfr*^*5304*^ flies) exhibits a reduced morning peak and a phase advanced evening peak in LD as well as a short period and a high percentage of arrhythmic flies in DD (Hyun et al., 2005; Lear et al., 2005) (Figure 5A, left). Although a large number of circadian cells including M and E cells normally express PDFR (Im et al., 2011), we could restrict PDFR expression to only the 5 Dv-E cells of *pdfr*^*5304*^ flies with the *DvPdf-GAL4, Pdf-GAL80* driver expressing PDFR in the *pdfr*^*5304*^ background. Essentially all major circadian deficiencies were rescued (Figure 5A, middle compared to 5A left and Table 1). Because CRY and PDFR are co-expressed in the same groups of circadian cells (Im et al., 2011), we inhibited PDFR expression in CRY-positive Dv-E cells with *Cry-GAL80*. This almost completely eliminated the rescue of the morning, evening peaks and rhythmicity on the *pdfr*^*5304*^*; DvPdf-GAL4, Pdf-GAL80/UAS-PDFR* flies (Figure 5A, right compared to Figure 5A, middle and Table 1), suggesting that it is the CRY-positive subset of Dv-E cells that are the key targets of PDF and that makes the major contribution to circadian behavior in LD as well as DD.

To localize these CRY-positive Dv-E cells, we stained *DvPdf-GAL4, Pdf-GAL80>UAS-mCD8:GFP* brains with an anti-CRY antibody and identified a single LNd (Figure 5B, arrow) as well as the 5^th^ small LNv ([Yoshii et al., 2008] and data not shown). To confirmed that there is only 1 CRY-positive LNd, adding a copy of *Cry-GAL80* to *DvPdf-GAL4>UAS-mCD8:GFP* flies eliminated GFP expression in that single LNd and the 5^th^-sLNv (Figure 5C; arrow points to a fiber from CRY+ LNd to the accessory medulla [Yoshii et al., 2008]) but not in M cells (Figure 5C). Based on the behavior (Figure 5A), these 2 CRY+ Dv-E cells play a more important circadian and activity role.

We next assayed the importance of PDFR expression in Dv-E cells for firing-induced phase-shifting. Although flies without PDF are too arrhythmic to assay phase-shifting (data not shown), the strain without the cognate receptor (*pdfr*^*5304*^ flies) is for unknown reasons a bit more rhythmic (30–60% rhythmicity) (Lear et al., 2009; Im et al., 2011), sufficient to support a phase-shifting assay. We therefore assayed firing-induced phase-shifting in rhythmic *PDFR* mutant groups with and without PDFR rescue in the 5 Dv-E cells.

The magnitude of the phase-shift was severely inhibited in *pdfr*^*5304*^ flies, consistent with the importance of PDF signaling for communication (Figure 5D), and expressing PDFR and dTrpA1 with the *DvPdf* driver rescued both phase delays and advances. Although the same experiment with the Dv-E cell driver (*DvPdf-GAL4; Pdf-GAL80*) failed to induce a phase-shift, the negative result probably reflects the requirement for dTrpA1 expression within PDF cells, which is inhibited by the presence of the *Pdf-GAL80* transgene. Also because rescue of PDFR expression in PDF cell and CRY negative Dv-E cells with *DvPdf-GAL4; Cry-GAL80* driver is not sufficient to rescue the major circadian deficiency (Figure 5A; Table 1; Lear et al., 2009) and phase-shifting of the PDFR mutant strain (Figure 5D), we favor the interpretation that PDFR expression in the 5 Dv-E cells, especially in the 2 CRY+ Dv-E cells, is sufficient to rescue PDF cell firing-induced phase-shifting like its rescue of circadian behavior (Figure 5C). The data taken together suggest that firing of morning cells releases PDF and that a subsequent association with its cognate receptor on Dv-E cells is important for both phase delays and advances.

### M cell firing induces CRY-independent phase shifts and TIM degradation

**Figure 6.**
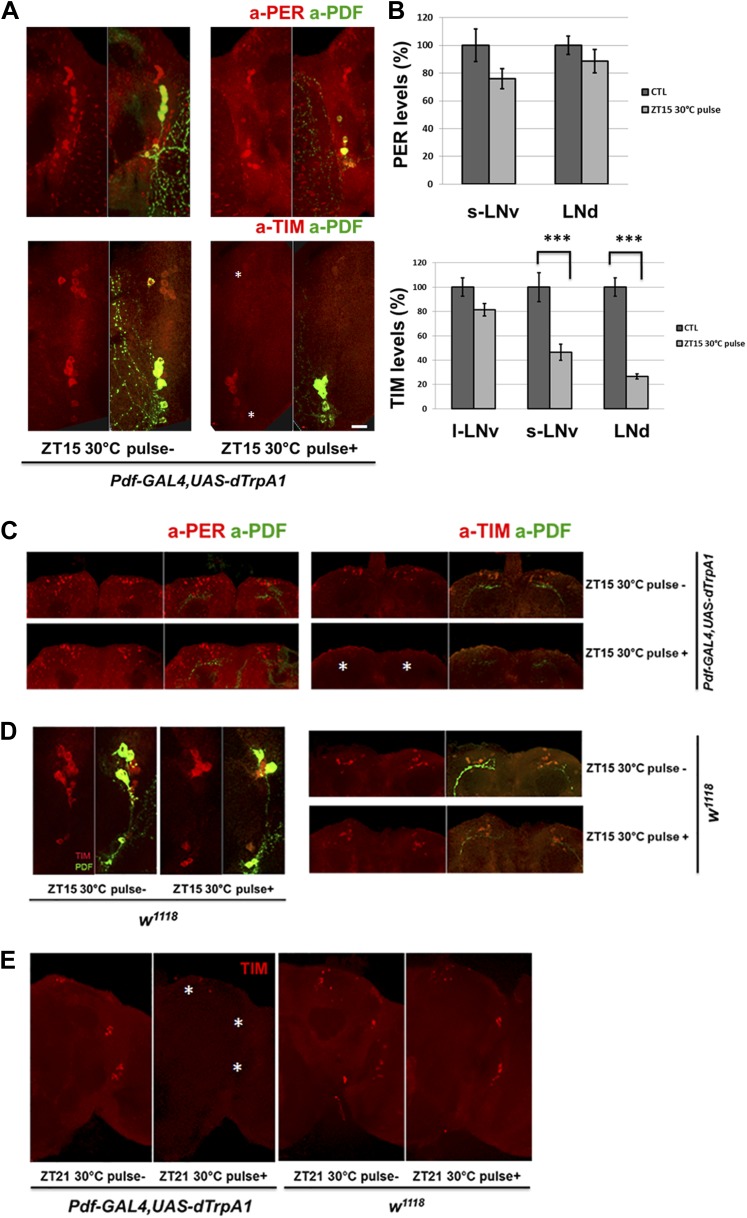
TIM but not PER in downstream circadian neurons responds to M cell firing. (**A**) TIM and PER staining in central pacemakers of fly brains after firing of M cells at ZT15. PER or TIM staining intensity is measured in *pdf-GAL4/UAS-dTrpA1* flies after a 2 hr 30°C pulse at ZT15. Brains were co-staining with anti-PDF (green) to visualize PDF+ cells. Asterisk indicates LNds (top) and s-LNvs cells (bottom). Note that staining in l-LNvs (higher than asterisk) does not change very much with firing. (**B**) Quantification of PER and TIM staining intensity in each group of clock neurons with or without a 30°C pulse (standard error of the mean [±SEM]). 5 brains and 10 hemispheres were quantified in each group. Scale bar = 20 μm. (**C**) TIM and PER staining in dorsal region (asterisk) of flies as described in (**A**). (**D**) TIM levels are not changed in wild-type fly brains after 2 hr at 30°C at ZT15. TIM levels in central (left panel) and dorsal circadian neurons (right panel) are shown in representative brains. (**E**) TIM levels are significantly decreased in *Pdf-GAL4/UAS-dTrpA1* brains (left panel) but not in WT control brains (right panel) after 2 hr 30°C pulse at ZT21. **DOI:** http://dx.doi.org/10.7554/eLife.02780.009

Because rapid CRY-dependent TIM degradation is a key event in light or heat-mediated phase-shifting (Suri et al., 1998; Yang et al., 1998), PDF cell firing might also trigger TIM degradation. There was indeed a dramatic and rapid reduction of TIM but not PER staining intensity in circadian neurons after M cell firing at ZT15 when TIM and PER are cytoplasmic (Figure 6A,B); the staining intensity of control flies without dTrpA1 expression was unaffected by the temperature shift (Figure 6D). M cell firing also caused nuclear TIM degradation in all circadian neurons at ZT21 (Figure 6E), consistent with the firing-mediated phase advances observed at this time (Figure 4B).

To complement the in vivo firing, we assayed the effect of in vitro firing on TIM degradation using the ATP-gated P2X2 cation channel (Lima and Miesenbock, 2005; Yao et al., 2012). Brains were dissected from *Pdf-GAL4; UAS-p2x2; cry*^*01*^ flies at ZT21 and incubated with 2.5 mM ATP for 2 hr. The inclusion of homozygous *cry*^*01*^ (null for CRY expression) in the genetic background was so that the brains would be light-blind, to minimize the effect of light on the TIM degradation assay.

**Figure 7.**
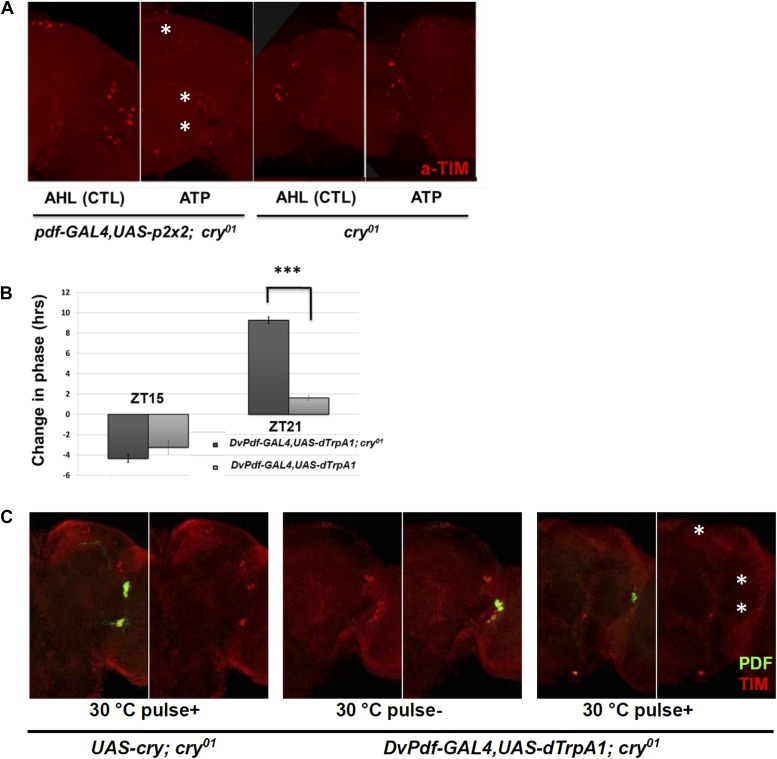
CRY is not required for M cell firing-induced TIM degradation and Phase Shifts. (**A**) TIM staining intensity strongly decreases in response to M cell firing caused by 2 hr incubation with 2.5 mM ATP (*Pdf-GAL4/UAS-p2x2; cry*^*01*^, left panel) at ZT21. TIM levels in the control *cry*^*01*^ group were not affected by the 2.5 mM ATP incubation (right panel). Asterisks indicate DNs, LNds and LNvs (top to bottom). (**B**) Firing-induced phase-shifting behavioral data in a *cry* null mutant strain. This strain shows a normal response to firing at ZT15 but an exaggerated response at ZT21. n = 32 for each group. ‘***’ represents p<0.001 as determined by the student's *t* test for normally distributed data. The error bars indicate SEM. (**C**) TIM is degraded in DNs, LNds and LNvs (asterisks, top and bottom, respectively) after firing at ZT21 even without CRY. An anti-PDF antibody (green) was used to visualize LNvs, and TIM was visualized with an anti-TIM antibody (red). TIM levels were markedly decreased after a ZT21 2 hr 30°C pulse of *DvPdf-GAL4/UAS-dTrpA1*; *cry*^*01*^ flies. TIM was not decreased in control *UAS-cry*; *cry*^*01*^ flies. The control strains are in the left four panels and the firing panels in the right two panels. Odd panels show staining with PDF and anti-TIM, whereas the even panels show staining only with anti-TIM. **DOI:** http://dx.doi.org/10.7554/eLife.02780.010

TIM levels were dramatically reduced after ATP application, and there was no significant effect in control *cry*^*01*^ flies (Figure 7A). The results mimic the similar effects of in vivo dTrpA1 activation, confirming that PDF neuron firing causes TIM degradation.

The canonical cell-autonomous model for TIM degradation and phase-shifting requires functional CRY (Emery et al., 2000). However, the in vitro P2X2 results suggest that firing-induced phase-shifting is CRY-independent. We therefore repeated the in vivo dTrpA1 experiments in a *cry*^*01*^ background.

Light pulses failed to change the phase of these *DvPdf-GAL4, UAS-dTrpA1; cry*^*01*^ flies as expected from the role of CRY in photoreception (data not shown), but they still responded to M cell firing. Although ZT21 phase advances showed a dramatic and enigmatic 10-hr advance in this background (Figure 7B), ZT15 phase delays in the *cry*^*01*^ background were only marginally different from wild-type. This suggests that delays may be more simply firing-dependent than advances, which may respond to a more complicated combination of light and firing.

We also examined TIM levels in these *cry*^*01*^ brains. Like the results in a wild-type strain, the pacemaker firing paradigm dramatically reduced TIM levels in these circadian neurons at ZT21, especially in the LNds; this further confirms that the neuronal firing-phase resetting pathway does not require CRY (Figure 7C). Our data taken together indicate that neural firing causes TIM degradation and phase resetting, which are controlled by a mechanism at least partially independent of CRY-mediated protein degradation.

### The ubiquitin ligase CUL-3 is involved in neuronal firing-mediated cytoplasmic TIM degradation

**Figure 8.**
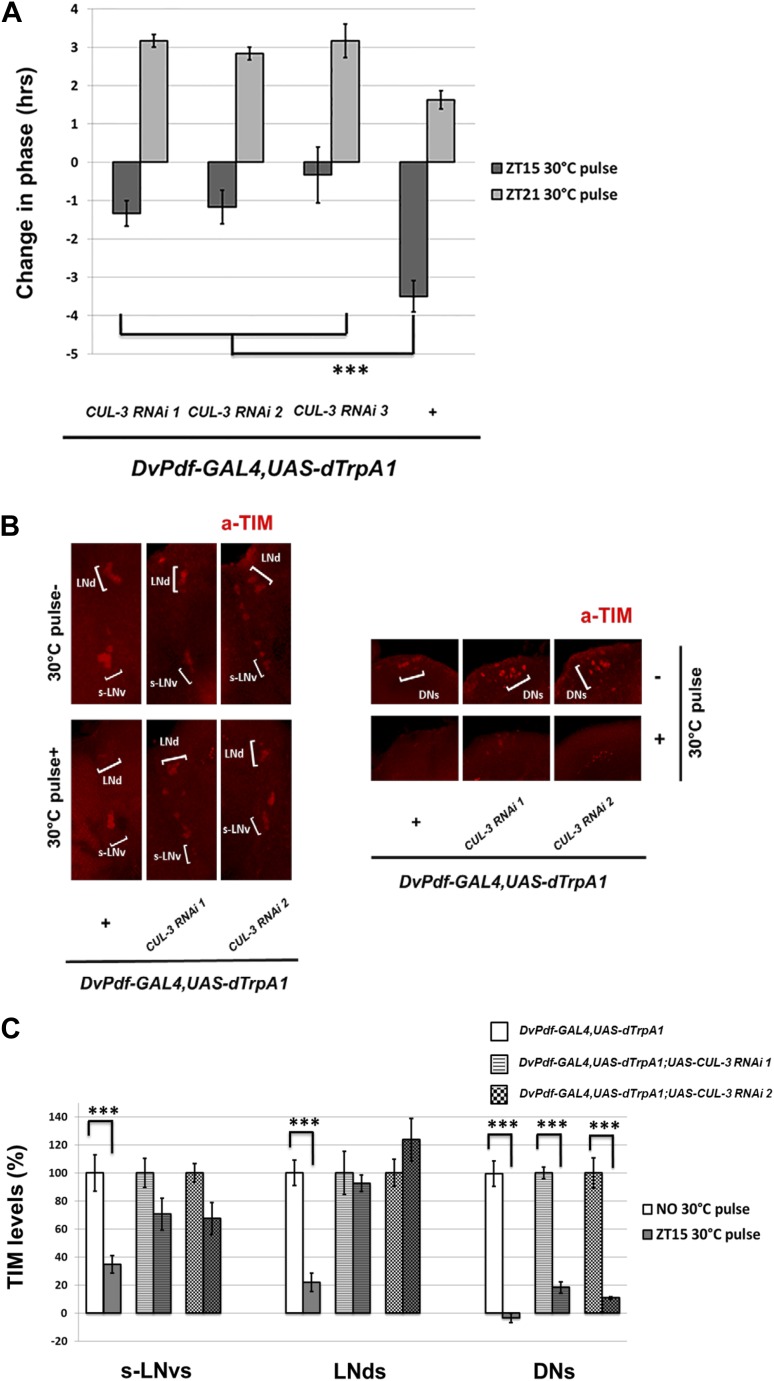
CUL-3 is involved in the delay zone phase shift response to PDF cell activation. (**A**) *CUL-3 RNAi* lines and background control lines were co-expressed with *dTrpA1* under control of *DvPdf-GAL4*. Their phase-shifts were measured after 2 hr 30°C pulse at ZT15 or ZT21. Genotypes were shown below each group. n = 30–32 for each group. ‘***’ means p<0.001 as determined by one way analysis of variance (ANOVA), Tukey post hoc test, and the error bars indicate SEM. (**B**) TIM levels were much less affected by neuron firing in DvPdf positive neurons expressing *CUL-3 RNAi*. Flies were transferred to 30°C and pulsed at ZT15 for 2 hr. Their brains were then dissected and immunostained with antibodies against TIM (red) and PDF (not shown). Different groups of circadian neurons were imaged and classified by their positions relative to PDF staining. Individual representative brains are shown. The experiment was repeated three times with qualitatively identical results in all three groups: LNds, s-LNvs and DNs. (**C**) TIM levels in different clock neuron groups for the genotypes described in (**A**) were quantified and normalized to the values of the control (same genotype without 30°C pulse, set to 100%). 10–12 hemispheres were examined in each group. ***p<0.001 compared to the controls as determined by the student's *t* test. The error bars indicate SEM. **DOI:** http://dx.doi.org/10.7554/eLife.02780.011

**Figure 9.**
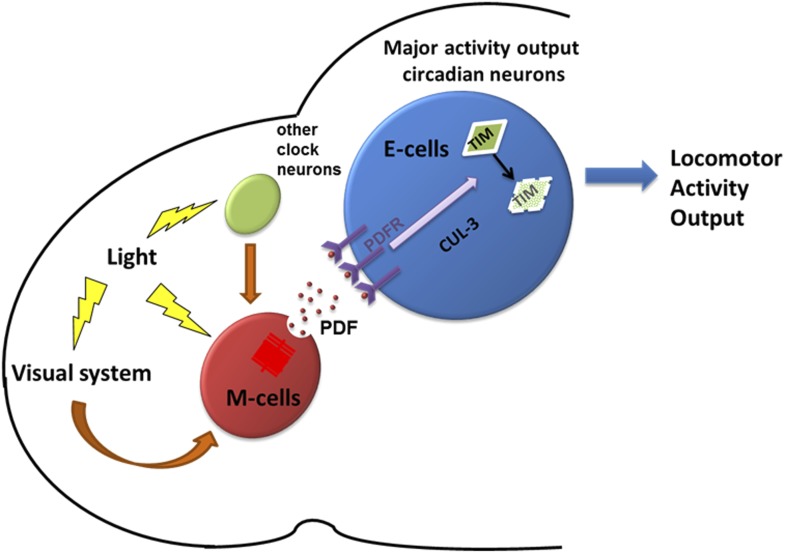
A model depicting how PDF and PDF-positive morning cells respond to light cues and control the pace of downstream E cells. Light activates morning cells directly or only indirectly through the visual system or from other clock neurons and induces PDF secretion. PDF then acts on downstream evening cells to promote cytoplasmic TIM degradation through a CRY-independent and CUL-3 dependent pathway, which causes phase or period adjustment. **DOI:** http://dx.doi.org/10.7554/eLife.02780.012

The E3 ubiquitin ligase component Cullin-3 (CUL-3) was recently shown to regulate TIM degradation and promote TIM/PER oscillations in DD (Grima et al., 2012). Moreover, the effect of CUL-3 is particularly striking when TIM is cytoplasmic in the early night. This suggests that CUL-3 might be important for PDF mediated, CRY-independent TIM degradation and phase-shifting, especially in the early night-delay zone. Indeed, co-expression of CUL-3 RNAi with dTrpA1 under *DvPdf-GAL4* control did not significantly affect DD rhythmicity and period at 21°C (Table 1; Grima et al., 2012). However, these flies had reduced firing-mediated phase delays at ZT15 (Figure 8A). TIM staining revealed that the co-expression also substantially reduced TIM degradation in *DvPdf-GAL4* labeled M and E cells (Figure 8B,C). In contrast, TIM degradation within the DNs was potent and indistinguishable from control strains, presumably because the *DvPdf-GAL4* driver and UAS constructs are not expressed in the DNs (Figure 8B). This suggests that the effects of the CUL-3 RNAi constructs are principally cell-autonomous and that dTrpA1-mediated M cell-firing remains potent despite co-expression of the RNAi constructs. The results taken together indicate that M cell firing in the early night activates a PDFR-CUL-3 pathway to reduce cytoplasmic TIM accumulation. The results also imply that TIM levels within the *DvPdf-GAL4-*labeled pacemakers make a substantial contribution to the magnitude of the phase delay. We therefore suggest that E cells are major pacemakers under more natural L-D conditions and that M cells serve principally to integrate light information and phase adjust the E cells through firing and PDF release (Figure 9).

## Discussion

Diurnal behavior in *Drosophila* is currently best explained by a dual-oscillator model, which emphasizes the M cells as master pacemakers and the E cells as secondary slave oscillators. Here we provide a modified configuration with a more prominent role for 5 Dv-E cells (Figure 9). This view is based on several new and unanticipated results. First, the 5 Dv-E cells are not only responsible for the evening anticipation peak and its timing, but they also contribute to the morning peak, free running rhythms and total activity. Otherwise put, these cells appear to play a key role in all aspects of circadian rhythms and locomotor activity. Second, E cell resetting can occur in a non cell-autonomous manner, through M cell neuron firing, PDF release and E cell PDFR activation. Third, TIM level changes within the 5 Dv-E cells in response to M cell firing likely make a major contribution to the resulting phase changes, at least in the early night when phase delays occur, and therefore make a major contribution to circadian timekeeping under more normal LD conditions.

The classical cell-autonomous model of *Drosophila* phase-shifting has long contrasted with the well-established view of phase-shifting in the mammalian SCN. In this system, firing from optic track activates SCN NMDA receptors, which is necessary and sufficient for light-mediated phase shifts. Importantly, the contribution of PDF cell firing and PDF to *Drosophila* phase-shifting brings this system closer to that of mammals. Because many aspects of Vasoactive intestinal polypeptide (VIP) function in mammals resemble those of PDF in flies (Vosko et al., 2007), it will be interesting to know if VIP cell firing can mimic light and phase-shift mammalian rhythms.

An important contribution of the E cell TIM level change to phase delays is consistent with previous work showing that a full light pulse at ZT15 induces rapid and potent TIM degradation in all 6 LNds as well as reduced TIM degradation in response to a light pulse of decreased intensity (Tang et al., 2010). This conclusion is also consistent with more recent data from Emery et al. (Lamba et al., 2014). Our data here also point to a more important role of the LNds rather than the M cells: the LNds show more TIM degradation in response to firing and a bigger response to the CUL-3 knockdown, which correlates with the decrease in phase delay caused by the knockdown (Figure 8).

This previous work also indicates that there is no observed TIM degradation in the PDF-positive M cells after a light pulse (Tang et al., 2010). This is consistent with older work showing that *cry* rescue in non M cell circadian neurons (with *Tim-GAL4/Pdf-GAL80*) provides a robust light mediated PRC (Stoleru et al., 2007), suggesting that canonical CRY-JET-mediated TIM degradation within M cells is unnecessary to convey light information to the brain clock. Our data extend this picture to the more dorsal circadian DNs by showing that TIM degradation within this circadian subpopulation is probably also not essential, at least to phase delays (Figure 8B,C) (Lamba et al., 2014). Although these data do not exclude a more inclusive view, our results indicate that acute PDF cell activation causes TIM degradation within the 5 Dv-E cells and that this degradation correlates with the magnitude of the phase delay. We note however that PDF cell activation may also function in other ways, for example by chronically affecting PER levels (Li et al., 2014) or by activating other downstream neurons (Seluzicki et al., 2014).

CUL-3 is the first ubiquitin ligase component other than JET to connect with phase-shifting. Importantly, CUL-3 has been shown to participate in light-independent TIM degradation within the cytoplasm (Grima et al., 2012). This is where TIM is localized during its accumulating phase in the early night (see Figure 6A), which perfectly matches the preferential role of CUL-3 in promoting delay zone phase-shifts.

However, the CUL-3 RNAi effects are incomplete. The new non cell-autonomous pathway described here may therefore cooperate with the more traditional cell-autonomous light-CRY-JET pathway to effect phase shifting. As the latter is essential for light-mediated phase-shifting, another non-exclusive possibility is that the two pathways are in series rather than in parallel, that is, that the light-CRY-JET pathway is upstream of neuronal firing and that the RNAi effects reflect incomplete knockdowns. This notion fits with recent studies suggesting that the light-CRY interaction promotes neuronal firing (Fogle et al., 2011).

At least 2 and probably 3 LNds are CRY-negative by antibody staining criteria even after many days of extended incubation in DD (Yoshii et al., 2008). As all 6 LNds respond similarly to a full light pulse and to neuronal firing, this indicates that TIM degradation within the CRY-negative LNds is non cell-autonomous and probably requires communication from M cells and/or from other neurons like their CRY-positive LNd neighbors (Lamba et al., 2014). If the CRY-negative LNds are also PDFR-negative (Im et al., 2011), other neuropeptides, neurotransmitters or perhaps even gap junctions may participate in the transfer of light or firing information upstream of TIM degradation.

Surprisingly, output from the 5 Dv-E cells controls a substantial fraction of evening activity as well as overall locomotor activity (Figure 2). These conclusions are also based on the phenotypes of Kir as well as dTrpA1 expression under *Dv-E GAL4* control (Figure 2B and data not shown). As *Dv-E GAL4/UAS-TNT* flies have significantly impaired activity in DD as well as in LD (Figure 2A), the residual locomotor activity may be clock-independent. However, these flies still manifest an evening anticipation peak under LD conditions (Figure 2A), suggesting incomplete suppression by TNT or a role of other circadian activity neurons. As the circadian activity-promoting subset of circadian neurons expresses CRY and PDFR and the *Dv-E GAL4* driver only labels 1 CRY positive LNd (Figure 2), a simple explanation is that one or more of the other 2 CRY^+^ LNds also promotes locomotor activity.

The Dv-E cells also contribute to morning activity. This is because rescue of PDFR expression only in the Dv-E cells is sufficient to rescue the morning anticipation peak (Figure 5C). In addition, Dv-E cell output is necessary for a robust morning peak (Figure 2A,B). Yet previous results connect the M cells to morning activity (Choi et al., 2012). These cells also communicate via PDF with the DN1p dorsal neurons, which also impact the morning anticipation peak (Zhang et al., 2010a, 2010b). Although all of these considerations suggest that redundant circuits downstream of the M cells underlie the morning anticipation peak, only output from the Dv-E cells has been shown to be necessary for morning activity, and there is evidence that even the M cells are not necessary for the morning activity peak (Sheeba et al., 2010).

These PDF+ M cells have been considered the master pacemakers based on their critical role in keeping time in constant darkness. However, their firing-resetting properties, the identification of the Dv-E cells as major activity neurons even in DD and the likely importance of TIM degradation within these cells for phase adjustment suggest a different reason for the strong molecular rhythms of the PDF neurons in DD and their contribution to behavioral rhythmicity under these conditions. These features likely reflect the important role of the PDF neurons as light-sensitive cells, directly through CRY and indirectly as post-synaptic targets of other light-sensitive cells such as photoreceptor cells within the eye and dorsal brain (Malpel et al., 2002; Yoshii et al., 2008 and unpublished data). We therefore suggest that a major function of the PDF neurons is to integrate light information and use it to phase-adjust the Dv-E cells, which are important pacemakers under LD conditions (Figure 9). Phase is a more important parameter than period under these more ‘natural’ conditions, and expression of period-altering mutants under Dv-E control appropriately alters the phase of the major evening peak but fails to do so under PDF control (Figure 1D and data not shown).

Lastly, our data offer a simple mechanistic explanation for a long-standing difference between peripheral and central circadian oscillators in *Drosophila*. The amplitude of peripheral oscillators is dependent on light as they damp rapidly after transfer of flies to constant darkness. Fly peripheral oscillators are also dependent on the photoreceptor CRY (Stanewsky et al., 1998; Malpel et al., 2002). In contrast, the circadian brain network and resultant rhythmic behavior persist in constant darkness and in the absence of CRY (Emery et al., 1998, 2000). Our work here can explain this difference in a simple way: neuronal firing replaces the function of light and CRY and even acts at the same step in the circadian cycle, namely, to promote TIM degradation within circadian neurons. We suggest that this step is intrinsically weak and that TIM degradation is normally potentiated every day, by light and/or by firing. This ensures the maintenance of robust rhythms of brain and behavior without compromising the light- and firing-sensitivity needed for phase adjustment.

## Materials and methods

### Fly strains

*DvPdf-GAL4* was provided by Dr. JH Park; *per*^*0*^*;UAS-per* was from Dr. Francois Rouyer; *UAS-dTrpA1* was from Dr. Paul Garrity; *pdfr*^*5304*^*; UAS-PDFR* were from Dr. Paul Taghert; *pdf-GSG* was from Dr. Fernanda Ceriani ; the *cry*^*01*^ mutant was from Dr. JC. Hall; *UAS-TNT* and *UAS-Tet* were from Dr. Hubert Amrein; UAS-DBT^s^ was from Dr. Jeffrey Price; *UAS-SGG; Pdf-GAL4, Pdf-GAL80, Cry-GAL80* were described by Stoleru et al. (2004); *UAS-p2x2* was from Dr. Orie Shafer; *UAS-CUL-3 RNAi 1* (VDRC 25875), UAS-*CUL-3 RNAi 2* (VDRC 109415), *UAS-PDF RNAi* (VDRC 4382), *UAS-Trh RNAi* (VDRC 105414) were from VDRC. *UAS-CUL-3 RNAi 3* (BL 36684) was from Bloomington stock center. All of the other GAL4 and UAS lines were obtained from the Bloomington stock center. Flies were reared on standard cornmeal/agar medium supplemented with yeast. The adult flies were entrained in 12:12 light–dark (LD) cycles at 25°C. The flies carrying *GAL4* and *UAS-dTrpA1* were kept at 21°C to inhibit dTrpA1 activity.

### Locomotor activity and statistical analyses

Locomotor activity of individual male flies (aged 3–7 days) was measured with Trikinetics Activity Monitors (Waltham, MA) for at least 4 days under 12:12 LD conditions followed by at least 7 days of constant darkness. The period and rhythmicity analysis was performed with a signal-processing toolbox implemented in MATLAB (MathWorks, Natick, MA) as described by Stoleru et al. (2004). Group activity was also generated and analyzed with MATLAB. For neuronal firing- induced phase-shift experiments, flies were entrained in LD for 4 days at 21°C, transferred to 30°C for 2 hr at ZT15 or ZT21 in the last night of the LD cycle and were put back to 21°C for the following DD days. For RU486 experiments, flies were fed with normal food for first 2 LD days and then transferred to tubes containing 200 μg/ml RU486 food (mifepristone, Sigma, USA) for 5 days (2 LD days and 3 DD days). At CT0 of DD4, the flies were put back to normal food. The phase difference was calculated by comparing the phase of RU486 feeding group with the vehicle feeding group.

All statistical analysis was conducted using IBM SPSS software. The Wilks–Shapiro test was used to determine normality of data. Normally distributed data were analyzed with 2-tailed, unpaired Student's *t* tests or one way analysis of variance (ANOVA) followed by a Tukey–Kramer HSD Test as the post hoc test. Data were presented as mean behavioral responses, and error bars represent the standard error of the mean (SEM). Differences between groups were considered significant if the probability of error was less than 0.05 (p<0.05).

### In vitro activation assay

All of the experimental and control flies were in a *cry*^*01*^ background to avoid light-induced TIM degradation during brain dissection. Adult flies were maintained longer in LD, for at least 5 days of entrainment, because of the *cry*^*01*^ background. Flies were collect and dissected in PBS at ZT21 when TIM levels are high. Fresh brains were incubated with AHL medium containing 2.5 mM ATP or vehicle for 2 hr and then fixed using the standard brain immunocytochemistry procedure.

### Fly brain immunocytochemistry

Immunostaining was performed as described (Tang et al., 2010). Fly heads were removed and fixed in PBS with 4% paraformaldehyde and 0.008% Triton X-100 for 45–50 min at 4°C. Fixed heads were washed in PBS with 0.5% Triton X-100 and dissected in PBS. The brains were blocked in 10% goat serum (Jackson Immunoresearch, West Grove, PA) and subsequently incubated with primary antibodies at 4°C overnight or longer. For TIM/PER/CRY and PDF co-staining, a rat anti-TIM (1:200), rabbit anti-PER (1:1000), rabbit anti-CRY (1:1000) and mouse anti-PDF (1:10) antibody (Developmental Studies Hybridoma Bank, University of Iowa, Iowa city, IA) were used as primary antibodies. For GFP staining, a mouse anti-GFP antibody (Invitrogen) was used at a 1:100 dilution. After washing with 0.5% PBST three times, the brains were incubated with Alexa Fluor 633 conjugated anti-rabbit (PER) and Alexa Fluor 488 conjugated anti-mouse (PDF) (Molecular Probes, Carlsbad, CA) at 1:500 dilution. The brains were washed three more times before being mounted in Vectashield Mounting Medium (Vector Laboratories, Burlingame, CA) and viewed sequentially in 1.1 μm sections on a Leica confocal microscope. To compare the fluorescence signals from different conditions, the laser intensity and other settings were set at the same level during each experiment. Fluorescence signals were quantified by ImageJ as described.
