# Supplementary material for: PDF neuron firing phase-shifts key circadian activity neurons in Drosophila
Source: eLife. 2014 Jun 17;3:e02780. doi: 10.7554/eLife.02780 (PMC4092873; doi:10.7554/eLife.02780)
Supplement: Related texts

# Article commentary: Decision letter

**DOI:** 10.7554/eLife.02780.013

**Affiliations:** University of California, San Francisco, United States

eLife posts the editorial decision letter and author response on a selection of the published articles (subject to the approval of the authors). An edited version of the letter sent to the authors after peer review is shown, indicating the substantive concerns or comments; minor concerns are not usually shown. Reviewers have the opportunity to discuss the decision before the letter is sent (see review process). Similarly, the author response typically shows only responses to the major concerns raised by the reviewers.

Thank you for sending your work entitled “PDF Neuron Firing Phase-shifts Key Circadian Activity Neurons in *Drosophila* for consideration at *eLife*. Your article has been favorably evaluated by K (Vijay) Raghavan (Senior editor), a Reviewing editor, and 3 reviewers.

The Reviewing editor has assembled the following comments based on the reviews to help you prepare a revised submission.

This study by the Rosbash lab addresses the function of the so-called morning and evening cells in the Drosophila brain clock that controls activity-rhythms. In particular, the authors ask whether phase-shifting of the clock (synchronization to day-night cycles) involves neuronal firing and intercellular communication. Although cell-autonomous CRYPTOCHOME-dependent degradation of the TIMELESS clock protein is a key mechanism in molecular phase-shifting, previous work from the same lab has shown that non cell-autonomous resetting mechanisms also operate in the clock neuronal network. They show here that neuronal excitation of the PDF-expressing morning cells can induce both advances and delays of the behavioral rhythms, similarly to what light pulses do. These behavioral phase shifts are correlated with TIM degradation in morning and evening cells. TIM degradation does not require CRY and appears to rely upon the activity of CUL-3 ubiquitylation complexes for evening signals.

These are very interesting data that reveal new mechanisms for the synchronization of the clock, both at the cellular and molecular level. Some control quantifications should added to strengthen the conclusions and a few additional experiments would be welcome. One weakness is the first part of the paper (Figures 1-2) where the authors question the role of PDF-negative cells in the LD and DD behavior. The results are rather different depending on the tools that are used to alter these cells and this should be clarified or toned down.

Major points:

1) Characterization of the *Dvpdf-gal4* cells: the so-called 5 Dv-E cells should be better characterized, most importantly for their CRY expression status since “evening” LNds have mostly been described through CRY expression. Please do anti-CRY labeling to tell whether these cells or some of them are CRY-positive.

2) The effects of E cell manipulations on DD rhythms and activity is very interesting (Figure 1D). However, how do the authors reconcile these findings with previous results (including those from their own lab) showing that clocks in M cells are necessary and sufficient for DD rhythms? Is it the case that clocks in E cells are not essential in DD, but the cells themselves are? Or that the cells are not required either, but tinkering with the activity of E cells interferes with the rhythms generated by M cells? Related to this point, what is the effect of M (Pdf) cell manipulation on activity levels in DD?

3) To strengthen the case that the E cells are driving the phase of activity in LD, I recommend that the authors include the data demonstrating that period-altering transgenes are more effective in altering phase when expressed in E cells than M cells (currently mentioned as “data not shown” in the Discussion).

4) The manuscript shows nicely that expression of pdfr in M+E cells rescues M cell-induced phase-shifting in a *pdfr* mutant background. The argument that the expression in E cells is the relevant one makes sense, and I recognize that restricting expression of pdfr to E cells, while driving TrpA in M cells, could be complicated (requiring two driver systems). However, the authors should show that restricting expression to M cells is ineffective (again, currently mentioned as “data not shown”).

5) Figure 1D/E:Why do TNT and Kir expression in the Dv-E cells produce so different behavioral phenotypes in LD ? Please clarify/explain.

TNT: no morning activity and evening activity only mildly affected. DD rhythms strongly affected.

Kir: morning activity does not seem very different in controls and *DvPdf-gal4* flies at 27°C (quantification of anticipatory activity should be shown to claim that they are different), whereas evening activity is virtually absent.

DD results should also be shown for Kir.

The DD period of the different genotypes should be shown in a table with the relevant parameters (number of flies, rhythms' robustness parameter).

6) Figure 2: Same comment about rhythms' parameters for DD analyses.

I do not understand this experiment. Based on the published work, is not it expected that expressing Kir or to a less extent PDF RNAI in PDF cells would essentially kill the DD rhythms? Please clarify.

In addition, what is the basis for claiming that the absence of a perS-induced period shortening is the consequence of non-communication between M and E cells specifically ?

7) Figure 3: These are nice experiments to show that firing in M cells phase shifts the behavioral clock similarly to what light does.

The ZT21 firing experiment (Figure 3B) should also be done with E cells only to conclude that M cells are the main players.

8. Figure 4B: PDFR role in phase-shifting. As for Figure 3, E cells should be tested at ZT21.

In addition, TrpA+PDFR in only M cells should be added to the figure, since it's a key result to exclude the possibility that PDFR signaling in M cells is doing the job.

9) Figure 5B: Tim degradation at ZT15.

TrpA-induced TIM degradation should be quantified in the DNs, as in Figure 5A for LNs.

The figure seems to show degradation in the DNs but the discussion indicates that DNs are not relevant for ZT15 pulses. Please clarify.

Figure 5D: Tim degradation at ZT21

A wt control with heat pulse should be added and a quantification of TIM degradation should be provided as in Figure 5A. It looks like TIM degradation is stronger at ZT21 than at ZT15. Is this true ?

10) Figure 6B. Phase shift in cry0 flies.

As indicated by the authors, cry0 flies display a spectacular and surprising phase advance. Could this be due to a change in TIM degradation induced by tim/jet alleles in the cry0 line (even through a cry-independent mechanism?). Since TIM degradation seems stronger for ZT21 experiments, the effects of the genetic background might be different for ZT15 and ZT21 responses. Do cry+/cry0 flies have the same tim/jet genotype ? How about TIM level in the absence of light ?

11) Figure 6C. TIM degradation in cry0 flies.

It seems that anti-TIM labeling disappears after the ZT 21 pulse in l-LNvs, whereas it does not change in a cry+ background (Figure 5D). This should be quantified.

The same experiment should also be done at ZT 15 and compared to cry+ flies in Figure 5A

12) Figure 7B. Cul-3 role in TIM degradation.

Since Cul3 inhibition does not prevent TIM degradation after a ZT21 pulse, it would be interesting to do the experiment in conditions where jetlag is inhibited

# Reply: Author response

**DOI:** 10.7554/eLife.02780.014

We thank all of the referees for the helpful comments. To address the most important ones, we added three major additions to our paper:

1) We added a panel to Figure 1 (now 1D) to show that the expression of a period-altering gene in E cells but not in M cells or in DN1p cells can change the activity phase in LD. This indicates that the E cell clock directs the phase of behavioral output.

2) We did a new experiment, shown in Figure 2B, addressing the effect of blocking E cell activity. This was previously done under incomplete conditions (26-27°C), and we have now done it at 30°C to effect a more complete block of E cell activity. As predicted there is a nearly complete loss of morning activity (as well as a strong effect on evening activity). Importantly, the two approaches, in 2A and 2B, are now much more similar than before. More than one referee had trouble with the previous difference between 2A and 2B, which is now essentially gone.

3) We did CRY staining in Figure 5 and now show that 1 of the Dv-LNds as well as the 5^th^ sLNv are CRY-positive. We also show behavior data to indicate that these 2 CRY+ Dv-E cells are key for the circadian phenotype.

*1) Characterization of the* Dvpdf-gal4 *cells: the so-called 5 Dv-E cells should be better characterized, most importantly for their CRY expression status since “evening” LNds have mostly been described through CRY expression. Please do anti-CRY labeling to tell whether these cells or some of them are CRY-positive*.

Thank you for the suggestion. We did CRY staining and also CRY-GAL80 behavior experiments and show the data in Figure 5. They are: 1) Only one of the four LNd cells is CRY positive (as well as the 5th s-LNv). 2) These CRY+ DV-E cells were essential for circadian behavior, i.e., adding CRY-gal80 to the PDFR rescue with the DV-E driver blocks the rescue of the M/E peaks on the PDFR mutant background.3) Conclusion: It is the 2 CRY+ cell that are most important, one or both.  And this doesn't exclude the other two CRY+ LNds as being effective too.

*2) The effects of E cell manipulations on DD rhythms and activity is very interesting (**Figure 1D**). However, how do the authors reconcile these findings with previous results (including those from their own lab) showing that clocks in M cells are necessary and sufficient for DD rhythms? Is it the case that clocks in E cells are not essential in DD, but the cells themselves are? Or that the cells are not required either, but tinkering with the activity of E cells interferes with the rhythms generated by M cells? Related to this point, what is the effect of M (Pdf) cell manipulation on activity levels in DD?*

In a nutshell, we think that the basic DD result is compatible with a model where E-cells are downstream of PDF cells. We think the activity phase is both controlled by the clock within the E cells (please see the answer to next comment) and PDF signaling from M cells and that the output from E cells is necessary to translate the PER/TIM phase within the PDF neurons into circadian behavior.

*3) To strengthen the case that the E cells are driving the phase of activity in LD, I recommend that the authors include the data demonstrating that period-altering transgenes are more effective in altering phase when expressed in E cells than M cells (currently mentioned as “data not shown” in the Discussion)*.

Please see addition to Figure 1, i.e., 1D. We have expressed DBT-S in M-cells, DN1ps and E-cells, and only DBT-S in E-cells moves the major activity peak in the expected direction. This is also compatible with the literature, where SGG expression was used to advance the evening peak (2007 Stoleru Cell paper). Our current paper extends that result by having a much more restricted expression pattern, and suggests that clock in E cells directly controls the major activity phase in LD. In other words, PDF acts through Dv-E cells to modulate phase and period.

*4) The manuscript shows nicely that expression of pdfr in M+E cells rescues M cell-induced phase-shifting in a* pdfr *mutant background. The argument that the expression in E cells is the relevant one makes sense, and I recognize that restricting expression of pdfr to E cells, while driving TrpA in M cells, could be complicated (requiring two driver systems). However, the authors should show that restricting expression to M cells is ineffective (again, currently mentioned as “data not shown”)*.

We added these data to Figure 5D. Since one copy of Cry-GAL80 (3^rd^ chromosome) only blocks GAL4 activity in CRY+ Dv-E-cells (1 CRY+ LNd as well as 5^th^ sLNv,) but not in M cells (Figure5C), we use *DvPdf-GAL4;Cry-GAL80* to drive the TrpA1 and PDFR expression in M as well as CRY negative Dv-E cells, which is ineffective to cause a M cell-induced phase-shifting in a *pdfr* mutant background.

*5)* *Figure 1D/E**:Why do TNT and Kir expression in the Dv-E cells produce so different behavioral phenotypes in LD ? Please clarify/explain*.

*TNT: no morning activity and evening activity only mildly affected. DD rhythms strongly affected*.

*Kir: morning activity does not seem very different in controls and* DvPdf-gal4 *flies at 27°C (quantification of anticipatory activity should be shown to claim that they are different), whereas evening activity is virtually absent*.

*DD results should also be shown for Kir*.

*The DD period of the different genotypes should be shown in a table with the relevant parameters (number of flies, rhythms' robustness parameter)*.

We redid the tub-kir experiment at 30 °C, because we suspected that the 27 °C used in paper does not induce full KIR expression in E cells. Indeed, we now show these stronger data in Figure 2B. We also include the suggested table.

*6)* *Figure 2**: Same comment about rhythms' parameters for DD analyses*.

*I do not understand this experiment. Based on the published work, is not it expected that expressing Kir or to a less extent PDF RNAI in PDF cells would essentially kill the DD rhythms? Please clarify*.

There is no conflict with Ceriani’s result. We of course chose only rhythmic flies to analyze; otherwise a phase shift cannot be determined. Then we only “opened” the driver for 3-4 days during DD, by feeding food for that length of time. Consistent with the Ceriani result, we recovered rhythmicity after transferring the flies from RU+ food back onto normal food.

In addition, what is the basis for claiming that the absence of a perS-induced period shortening is the consequence of non-communication between M and E cells specifically ?

We clarified the prose and now say that this failure fulfills a prediction of our model, that without PDF and firing there is no communication from M cells.

*7)* *Figure 3**: These are nice experiments to show that firing in M cells phase shifts the behavioral clock similarly to what light does*.

*The ZT21 firing experiment (**Figure 3B**) should also be done with E cells only to conclude that M cells are the main players*.

We have the data but for the sake of additional figures in this case would prefer to just say “data not shown” in the text, since we already have so many panels.

*8.* *Figure 4B**: PDFR role in phase-shifting. As for* *Figure 3**, E cells should be tested at ZT21*.

We have done this and now show the data as an additional bar in Figure 5D.

*In addition, TrpA+PDFR in only M cells should be added to the figure, since it's a key result to exclude the possibility that PDFR signaling in M cells is doing the job*.

This is the same comment as 4 above, and we added these data to Figure 5D.

*9)* *Figure 5B**: Tim degradation at ZT15*.

*TrpA-induced TIM degradation should be quantified in the DNs, as in* *Figure 5A* *for LNs*.

This could be done. However, we chose not to do it as the result is completely obvious, i.e., qualitative in nature. There is virtually no staining detectable after firing, and we could not even locate the DNs since the TIM levels in this part of the brain are equal to the background.

*The figure seems to show degradation in the DNs but the discussion indicates that DNs are not relevant for ZT15 pulses. Please clarify*.

Our explanation is based on the cul-3 RNAi result. We conclude that TIM in DNs is still degraded, whereas the phase shift and TIM degradation within the LNds are reduced.

*Figure 5D**: Tim degradation at ZT21*

*A wt control with heat pulse should be added and a quantification of TIM degradation should be provided as in* *Figure 5A**. It looks like TIM degradation is stronger at ZT21 than at ZT15. Is this true ?*

We added the WT control data to Figure 6E. As for the differences in degradation strengths at the two times, it is possible that they are slightly different (e.g., 80% in one case and 50% in the other). These small differences may be due to the use of different mechanisms, i.e., JET vs Cul3. We have chosen not to focus on these downstream mechanisms in this manuscript.

*10)* *Figure 6B**. Phase shift in cry0 flies*.

As indicated by the authors, cry0 flies display a spectacular and surprising phase advance. Could this be due to a change in TIM degradation induced by tim/jet alleles in the cry0 line (even through a cry-independent mechanism?). Since TIM degradation seems stronger for ZT21 experiments, the effects of the genetic background might be different for ZT15 and ZT21 responses. Do cry+/cry0 flies have the same tim/jet genotype ? How about TIM level in the absence of light ?

Although this is an interesting comment, we deliberately avoided focusing on cry/jet/TIM in this current paper. This is because we already have a large number of findings and because these issues are not central, i.e., they will divert attention from the current focus of our paper. The requested experiments are also labor-intensive. A new paper we have in mind will address the role of CRY.

*11)* *Figure 6C**. TIM degradation in cry0 flies*.

*It seems that anti-TIM labeling disappears after the ZT 21 pulse in l-LNvs, whereas it does not change in a cry+ background (fig 5D). This should be quantified*.

*The same experiment should also be done at ZT 15 and compared to cry+ flies in* *Figure 5A*

We did the ZT15 TIM staining in cry0 flies and the result is the same—TIM is still degraded after ZT15 pulse. However, we prefer not to show it but only mention it in text. We think the TIM levels in l-LNvs after pulse are interesting but not very related to central story of the paper.

*12)* *Figure 7B**. Cul-3 role in TIM degradation*.

Since Cul3 inhibition does not prevent TIM degradation after a ZT21 pulse, it would be interesting to do the experiment in conditions where jetlag is inhibited

We have these data but chose not to focus on them in this paper. Also, *jetlag* mutants have high CRY level, which can counteract PDFR signaling (preliminary behavior data). As mentioned above, we would like to avoid the roles of CRY and JET other than to show that there are strong and credible effects in the absence of CRY.
